# Prostaglandin E_2 _receptor subtype 2 (EP2) regulates microglial activation and associated neurotoxicity induced by aggregated α-synuclein

**DOI:** 10.1186/1742-2094-4-2

**Published:** 2007-01-04

**Authors:** Jinghua Jin, Feng-Shiun Shie, Jun Liu, Yan Wang, Jeanne Davis, Aimee M Schantz, Kathleen S Montine, Thomas J Montine, Jing Zhang

**Affiliations:** 1 Department of Pathology, University of Washington School of Medicine, Seattle, WA, USA

## Abstract

**Background:**

The pathogenesis of idiopathic Parkinson's disease (PD) remains elusive, although evidence has suggested that neuroinflammation characterized by activation of resident microglia in the brain may contribute significantly to neurodegeneration in PD. It has been demonstrated that aggregated α-synuclein potently activates microglia and causes neurotoxicity. However, the mechanisms by which aggregated α-synuclein activates microglia are not understood fully.

**Methods:**

We investigated the role of prostaglandin E_2 _receptor subtype 2 (EP2) in α-synuclein aggregation-induced microglial activation using *ex vivo, in vivo and in vitro *experimental systems.

**Results:**

Results demonstrated that ablation of EP2(EP2^-/-^) significantly enhanced microglia-mediated *ex vivo *clearance of α-synuclein aggregates (from mesocortex of Lewy body disease patients) while significantly attenuating neurotoxicity and extent of α-synuclein aggregation in mice treated with a parkinsonian toxicant 1-methyl-4-phenyl-1,2,3,6-tetrahydropyridine. Furthermore, we report that reduced neurotoxicity by EP2^-/- ^microglia could be attributed to suppressed translocation of a critical cytoplasmic subunit (p47-phox) of NADPH oxidase (PHOX) to the membranous compartment after exposure to aggregated α-synuclein.

**Conclusion:**

Thus, it appears that microglial EP2 plays a critical role in α-synuclein-mediated neurotoxicity.

## Background

Increasing evidence has suggested that neuroinflammation may contribute significantly to neurodegeneration in parkinsonian animals or even human Parkinson's disease (PD) [1-3]. One of the key features of neuroinflammation is microglial activation with resultant morphological changes, increased expression of cell surface receptors, and production of neurotrophic as well as neurotoxic factors [4]. The mechanisms underlying microglial activation in parkinsonian animal models or in human PD are largely unknown. Potential activators include environmental toxicants, e.g. rotenone and 1-methyl-4-phenyl-1,2,3,6-tetrahydropyridine (MPTP) [5,6], as well as endogenous substances, e.g. neuromelanin [7] More recently, we have demonstrated that aggregated α-synuclein, a major component of Lewy bodies in PD and associated synucleinopathies [8] potently activates microglia, leading to dopaminergic (DAergic) toxicity in part through activation of a membrane-associated nicotinamide adenine dinucleotide phosphate (NADPH) oxidase (PHOX) [9].

The processes involved in microglial activation and PHOX activation following aggregated α-synuclein exposure, however, are largely unknown. One clue to the relationship between microglial phagocytosis of protein aggregates and subsequent microglial activation comes from our recent investigation, where microglial activation induced by aggregated amyloid β (Aβ) is altered when a receptor for a prostaglandin E_2 _(EP2) is removed by genetic ablation. The results suggest that microglia derived from EP2^-/- ^mice appear to have increased capacity for clearance of Aβ peptides from tissue sections of patients with Alzheimer's disease (AD) via phagocytosis without the increased microglial-mediated paracrine neurotoxicity induced by Aβ [10]. Given that PGE_2, _a product derived from arachidonic acid by cyclooxygenase (COX) and specific synthases, is also significantly elevated in the substantia nigra (SN) and cerebrospinal fluid of PD patients [11], in this study we investigated whether EP2 played any role in the formation or handling of aggregated α-synuclein, events critically important in the pathogenesis of PD. This was achieved by using complementary approaches, including *ex vivo *experiments with human tissue, *in vivo *experiments with the parkinsonian toxicant MPTP, and finally *in vitro *experiments with purified microglia exposed to aggregated α-synuclein. Our results clearly demonstrated that microglial EP2 contributed to α-synuclein aggregation and associated neurotoxicity as well as microglial activation.

## Methods

### Materials

All chemicals were purchased from Sigma-Aldrich (St. Louis, MO) unless stated otherwise. α-Synuclein and neuronal nuclei (NeuN) antibodies were from Chemicon (Temecula, CA); CD11b was from Serotec (Raleigh, NC); p67^-phox ^and p47^-phox ^antibodies were from BD Bioscience (San Diego, CA). Alexa fluorescent-labeled secondary antibodies were from Molecular Probes (Eugene, OR). 4,6-Diamidino-2-phenylindole (DAPI)-containing mounting medium was from Vector Laboratories (Burlingame, CA). Papain and DNase I were from Worthington Biochemical (Lakewood, NJ). Culture media, heat-inactivated fetal bovine serum, and penicillin/streptomycin were from Invitrogen (Carlsbad, CA). Purified human α-synuclein was from r-Peptide (Athens, GA)

### Ex vivo studies

Frozen human mesocortex tissue from patients with Lewy body disease was used as a source of physiologically aggregated α-synuclein. Tissue slices were prepared exactly as described for similar studies using AD tissue as a source of physiologically aggregated Aβ [10,12]. All tissue was obtained from patients who died with Lewy body disease and who volunteered to donate brain tissue to the Neuropathology Core of the Alzheimer Disease Research Center at the University of Washington. All tissue was cryostat sectioned into 10 μm thick slices, mounted onto poly-D-lysine-coated coverslips, and placed in 24-well tissue culture plates as previously described by us and others [10,12].

EP2^-/- ^mice are a gift from Dr. Richard Breyer at Vanderbilt University Medical Center (Nashville, TN). Mice homozygous for disruption of the gene that encodes EP2(EP2^-/-^) were backcrossed >12 generations to the BALB/c genetic background [13]. Age-matched BALB/c wild-type (WT) control mice were obtained from Charles River Laboratories (Wilmington, MA). Mice were maintained in a temperature-controlled specific pathogen-free (SPF) facility with a strict 12-hour light/dark cycle and with free access to food and water. All experiments were performed exactly as approved by the University of Washington Institutional Animal Care and Use Committee (IACUC).

Primary microglia were isolated as described previously [9,14]. Briefly, microglia at 14^th ^day *in vitro *(DIV) were separated from the underlying astrocytic monolayer by gentle agitation using their differential adhesive properties and were seeded onto 10 micron mesocortical sections set in 24-well plates (described above) at 1 × 10^5 ^cells per section in microglial culture medium for 2 hr followed by an additional 48 hr incubation in serum-free DMEM containing penicillin and streptomycin. Following incubation, the contents of each well (microglia plus human tissue) was either lysed with 8 M urea and Western blotted for remaining α-synuclein aggregates, or fixed for immunohistochemical studies to visualize activated microglia and α-synuclein aggregates. For immunohistochemical analysis, fixed cultures (with 4% paraformaldehyde in PBS) were subjected to formic acid (88%) treatment prior to application of antibodies against CD11b (1:50) and anti-α-synuclein (1:200). Mounting medium containing DAPI was used to label the nuclei. Of note, to minimize the variation among sections used for WT and EP2^-/- ^microglia (see below), consecutive sections were selected.

### In vivo studies

For chronic MPTP treatment, 8- to 10-wks WT and EP2^-/- ^Balb/C mice, weighing 18–22 g at the beginning of the study, were rendered parkinsonian with a protocol previously used by us [15]. Briefly, mice were treated with 10 doses of MPTP hydrochloride (25 mg/kg in saline, s.c.) and the adjuvant probenecid (250 mg/kg in DMSO, i.p.) on a five-week schedule with an interval of 3.5 days between consecutive doses. Probenecid was used to inhibit the rapid clearance and excretion of MPTP from the brain and kidney following each injection. Control groups were treated with probenecid alone. Five weeks after the last treatment, the brains were rapidly removed and blocked sagittally with half fixed in freshly prepared paraformaldehyde and the other half dissected and stored at -70°C until assayed. DA concentration in each animal were quantified using HPLC-EC as described previously [15]. For sub-chronic MPTP treatment, mice received MPTP hydrochloride (30 mg/kg in saline, s.c.) once a day for 5 consecutive days; the animals were sacrificed five days after the last injection to measure striatal DA [16].

The method for fractionation of α-synuclein aggregates was described in our previous study [17]. Frozen mouse substantia nigra (SN) and striatum were homogenized in a NP40 lysis buffer containing 0.5% NP-40, 150 mM NaCl, 50 mM Tris, pH 8.0, 700 U/ml DNase I, and protease inhibitor cocktail. One small aliquot of cell lysate was removed for later total protein determination using the BCA assay (Pierce, Rockford, IL). An equal amount of homogenate was centrifuged at 10,000 × *g *for 10 min to yield a NP-40 soluble fraction and a pellet. The pellet was re-suspended in sodium dodecyl sulfate (SDS) buffer (2% SDS, 62.5 mM Tris, pH 6.8, 10% glycerol) and incubated at room temperature for 30 min with constant agitation. The extract was then centrifuged at 14,000 × *g *for 10 min at 4°C to generate an SDS-soluble (NP40-insoluble) fraction. Distribution of protein aggregates was compared in various experimental conditions between the two fractions by Western blot analysis.

### In vitro studies

To generate *in vitro *aggregated α-synuclein, purified human α-synuclein (1 mg/mL) was incubated in PBS at 37°C with constant agitation using a magnetic stir bar in 1.7 mL Eppendorf tubes. The resultant α-synuclein species, predominantly oligomers, are almost identical to those obtained by aging *in vitro *for 7 days without stirring [9]. Microglia (see above) were seeded in six-well plates at 1–2 × 10^6 ^cells per well in microglial culture medium, incubated overnight, and then exposed to *in vitro *aggregated α-synuclein for 30 min. After treatment, cells were washed twice with ice-cold PBS and then scraped off in a non-detergent homogenization buffer (250 mM sucrose, 10 mM Tris-HCl (pH7.8), 5 mM MgCl_2_, 2 mM EGTA, 2 mM EDTA, and protease inhibitor cocktail (1 mM PMSF). The cell lysate was centrifuged at 1000 × *g *for 10 min to remove cell debris and crude nuclei. Protein concentration of supernatant was measured by BCA assay. An equal amount of supernatant was further centrifuged at 55,000 rpm (Optima™ MAX benchtop ultracentrifuge, Beckman Coulter, Fullerton, CA) for 90 minutes to separate into cytoplasm (supernatant) and membrane (pellet) fractions. The pellets were resuspended in SDS lysis buffer (2% SDS, 62.5 mM Tris, pH 6.8, and 10% glycerol) in preparation for Western blotting to assess translocation of NADPH subunits (p47 and p67).

### Western blot

An equal volume of proteins from buffer- and SDS-soluble fractions (for analysis of α-synuclein aggregates distribution in mouse brain tissue) or from cytoplasm and membrane fractions (for analysis of NADPH subunits translocation) were diluted 1:2 in 2× loading buffer containing 5% β-mercaptoethanol and heated to 95°C for 10 min before loading onto 8–16% SDS polyacrylamide gels. Following separation, the proteins were transferred to PVDF membranes (Bio-Rad Laboratories, Hercules, CA), and probed overnight at 4°C with polyclonal rabbit anti-α-synuclein (1:5000) or p47-phox/p67-phox (1:1000) primary antibodies. After washing with TBST (0.1% Tween-20 in TBS), goat anti-rabbit-HRP secondary antibody was added at 1:40,000 for 1 h at room temperature, and detection was carried out by enhanced chemiluminescence. The relative intensity of the corresponding bands was quantified with Quantity One (Bio-Rad).

### Statistical methods

Repeated measures were performed at least three times in all experiments. Grouped data were expressed as mean ± S.E.M. Changes between two groups were analyzed by t-test or two-way analysis of variance (ANOVA), depending on the experiments, using a commercially available computer software program (Prism 3.0; GraphPad, San Diego, CA) with α = 0.05.

## Results

### EP2^-/- ^microglia enhanced the clearance of α-synuclein aggregates in the mesocortex of patients with Lewy body disease

**Figure 1:**
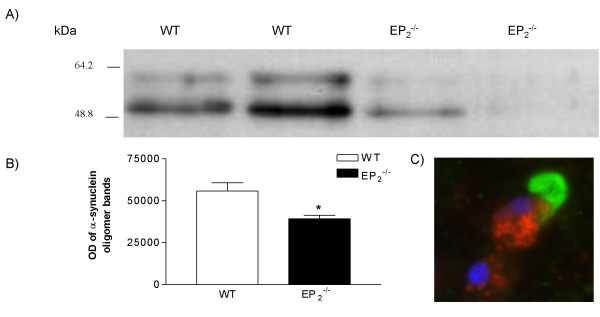
**Lack of EP2 enhanced microglial clearance of α-synuclein aggregates**. **A and B)**. Tissue sections obtained from patients with dementia with Lewy body disease were incubated with WT and EP2-/- microglia for 48 hrs. Residual α-synuclein aggregates were determined by Western blotting (two bands corresponding to dimers and trimers of α-synuclein). *: p < 0.05 for amount of residual synuclein aggregates in tissue sections treated with EP2-/- vs. WT microglia. **C) **Tissue sections were stained with CD11b antibody 48 hrs after incubation with microglia. The image demonstrates an activated microglial cell in ex vivo-cultured slides in close proximity to aggregated α-synuclein. Red, CD11b for microglia showing a macrophage-like microglia; green, antibody against human α-synuclein, demonstrating α-synuclein aggregates; and blue, DAPI nuclear staining).

We have previously demonstrated that microglia lacking EP2 show enhanced phagocytosis of Aβ aggregates while at the same time display suppressed bystander damage to neurons [10]. Using the technique of Bard et al. [12] in that study we incubated mouse microglial cultures with 10 micron tissue slices of human AD brain as a source of physiologically aggregated Aβ. To test whether these results can be extended to α-synuclein aggregates, we used a similar human *ex vivo *model. Primary microglia from either EP2^-/- ^or WT mice were incubated with mesocortical sections of patients with Lewy body disease as a source of physiologically aggregated α-syuclein oligomers for 48 hrs, followed by quantification of residual α-synuclein oligomers by Western blot analysis. The distribution and morphology of microglia in each experiment were also assessed microscopically after immunohistochemical staining against CD11b, a marker for activated microglia. Our results demonstrated that the level of α-synuclein oligomers was less in sections incubated with EP2^-/- ^microglia compared to consecutive sections incubated with WT microglia. A representative Western blot is shown in Figure 1A. Quantitative assessment (Figure 1B) indicated that the residual α-synuclein oligomers in the sections incubated with EP2^-/- ^microglia was approximately 70% of that incubated with WT microglia (P < 0.05; n = 6). A representative section incubated with EP2^-/- ^microglia is shown in Figure 1C, where activated microglia were identified in close proximity to aggregated α-synuclein.

### EP2^-/- ^mice were more resistant to neurotoxicity induced by MPTP

**Figure 2:**
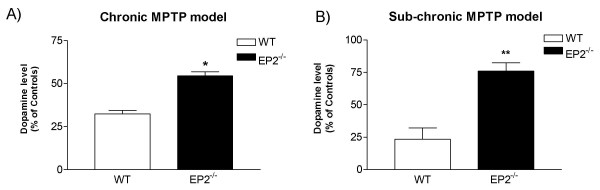
**Lack of EP2 suppressed loss of striatal dopamine in MPTP-treated mice**. Mice were treated with either a chronic (**panel A**) or sub-chronic regimen of MPTP (**panel B**). In the chronic protocol, mice were treated with MPTP (30 mg/kg × 10) and the adjuvant probenecid (250 mg/kg) on a five-week schedule with an interval of 3.5 days between consecutive doses. In the sub-chronic model, mice were treated with MPTP (30 mg/kg*day) or vehicle for five days. Remaining DA in the striatum of mice was measured by HPLC 5 weeks or 5 days post-final treatment in the chronic and subchronic models, respectively. Data are expressed as % control where control mice were the corresponding genotype treated with vehicle. *, **: p < 0.05 and p < 0.01 comparing EP2-/- with WT mice, respectively.

Given that microglia from EP2^-/- ^mice exhibited enhanced capacity to clear aggregated α-synuclein from human tissue, we next investigated the effects of ablation of EP2^-/- ^on nigrostriatal neurodegeneration induced by the parkinsonian toxicant MPTP using a chronic regimen that was established in our lab recently. In this model, EP2^-/- ^and WT mice were treated with MPTP at 25 mg/kg along with an adjuvant (probenecid), at 250 mg/kg for 5 weeks. Five weeks after the last treatment, the residual striatal DA level in each mouse was determined as an index of the extent of nigrostriatal damage. We found that there was no significant difference in DA levels in WT and EP2^-/- ^mice without MPTP treatment (P > 0.05, data not shown). The remaining striatal DA level in WT mice treated with MPTP was reduced to 32 ± 2% of the level in vehicle-treated WT mice (Figure 2A), similar to our previous results [15]. In contrast, the remaining striatal DA level in EP2^-/- ^mice treated with MPTP was 56 ± 2% of the level in vehicle-treated EP2^-/- ^mice (Figure 2A). We also tested whether ablating EP2^-/- ^had any effect in a sub-chronic model, where mice were treated with MPTP at 30 mg/kg per day for five days, and the extent of nigrostriatal degeneration was assessed five days after the last injection. It is remarkable that this sub-chronic model largely recapitulated what was observed in mice treated with MPTP chronically. In fact, the residual DA level was not significantly different between EP2^-/- ^mice treated with MPTP and those treated with vehicle, while WT mice lost > 75% of their striatal DA after MPTP treatment.

### Attenuated formation of NP-40 insoluble α-synuclein aggregates in EP2^-/- ^mice

**Figure 3:**
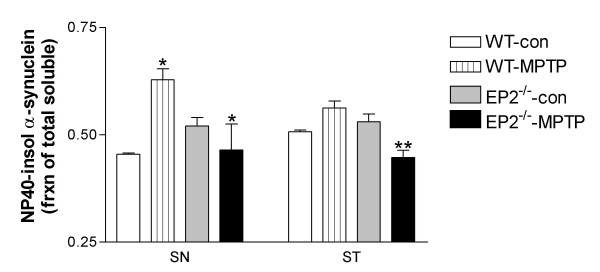
**Lack of EP2 attenuated formation of NP40-insoluble α-synuclein aggregates in both SN and striatum**. SN and striatum were dissected at 5 weeks after the last treatment and fractionated into NP40-soluble and NP40-insoluble/SDS-soluble fractions, followed by assessment by Western blot. Data is expressed as NP-40 insoluble (SDS-soluble) α-synuclein aggregates as a fraction of total (NP40- and SDS-) soluble α-synuclein. *: p < 0.05 comparing WT-MPTP vs. WT-control (con) and EP2-/--MPTP vs. WT-MPTP, respectively, in the SN. **: p < 0.01 comparing EP2-/--MPTP vs. WT-MPTP in the striatum (n = 8).

Reduced neurotoxicity in EP2^-/- ^mice exposed to MPTP could derive from several possible mechanisms. As α-synuclein aggregation plays critical roles in neurodegeneration in human PD as well as parkinsonian animals, we next tested the hypothesis that EP2^-/- ^mice had altered accumulation of detergent-soluble α-synuclein oligomers, precursors of insoluble α-synuclein fibrils and the putative major neurotoxic species of α-synuclein [18]. We focused on the chronic MPTP model as previous experiments have demonstrated that Lewy body-like inclusions can be induced in this model at later time points [15,19]. Both the substantia nigra (SN) and striatum were dissected and extracted into NP40-soluble and NP40-insoluble/SDS-soluble fractions. We determined by Western blot the relative distribution of α-synuclein aggregates between theses two fractions, expressed as percent of detergent-soluble α-synuclein aggregates (from both fractions) in the NP40-insoluble fraction (Figure 3). Several observations were made: 1) there was no significant difference in basal (vehicle-treated) distribution of α-synuclein aggregates between EP2^-/- ^and WT mice, whether in the SN or striatum; 2) the shift in distribution of detergent-soluble α-synuclein aggregates (from NP40-soluble to NP40-insoluble) in WT mice was statistically significant at five weeks post-MPTP treatment in the SN but not in the striatum; and 3) in contrast to WT mice, in EP2^-/- ^mice there was no significant change in the distribution of detergent-soluble α-synuclein aggregates between the two fractions in either the SN or striatum following MPTP treatment.

### Attenuated translocation of PHOX p47 subunit from cytoplasm to membrane in EP2^-/- ^microglia treated with α-synuclein oligomers

**Figure 4:**
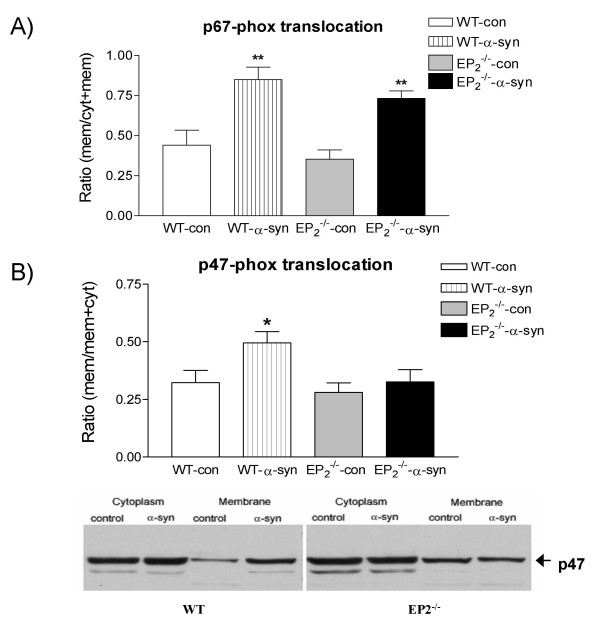
**Lack of EP2 reduced translocation of p47-phox but not p67-phox subunit from cytoplasm to membrane in microglia after α-synuclein oligomer treatment**. WT or EP2-/- microglia were seeded in 6-well plates overnight and then treated with aged α-synuclein or vehicle for 30 min. The cells were homogenized and fractionated into cytoplasm (cyt) and membrane fractions (mem). The relative distribution of p67-phox (panel A) and p47-phox (panel B) in the two fractions was analyzed with Western blot in order to assess translocation from the cytoplasm to the membrane (expressed as ratio of relative amount in membrane fraction to membrane + cytoplasm). *: p < 0.05, comparing EP2-/- with WT microglia. **: P < 0.01, comparing α-synuclein treatment with control.

Given our previous findings with EP2^-/- ^microglia and microglia-mediated bystander damage to neurons, another possible explanation for suppressed neurotoxicity in MPTP- exposed mice was reduced activation of neurotoxic components of the microglia response. Indeed, our previous experiments have demonstrated that extracellular aggregated α-synuclein potently activates microglia, leading to activation of PHOX (NADPH oxidase) and enhanced DAergic neurotoxicity [9]. PHOX activation involves translocation of several critical cytoplasmic units to the membrane compartment; thus, we next investigated whether ablation of the EP2^-/- ^receptor had any effects on two of these subunits, p67-phox and p47-phox. Microglia were isolated from WT and EP2^-/- ^mice, respectively, and seeded in 6-well plates overnight and then treated with pre-aged α-synuclein or vehicle for 30 min. The cells were then homogenized and fractionated into cytoplasmic and membrane fractions, and the fractions Western blotted. Translocation of p67-phox and p47-phox was analyzed by determining changes in their relative levels in the cytoplasmic and membrane fractions following exposure to aggregated α-synuclein. The results, presented in Figure 4, show that aggregated α-synuclein led to translocation of both subunits to membrane in WT glia. Intriguingly, while there was no significant difference in the translocation of p67-phox subunit (Figure 4A) between the two types of microglia, translocation of the p47-phox subunit appeared to be attenuated in EP2^-/- ^microglia treated with aged α-synuclein compared to WT controls. More specifically, approximately 50% of p47-phox was shifted into the membrane compartment in WT treated with aged α-synuclein, compared to approximately 30% of this subunit in EP2^-/- ^microglia (n = 5, p < 0.05).

## Discussion

Several major observations were made in this study, including: 1) microglia isolated from EP2^-/- ^microglia exhibited enhanced clearance of aggregated α-synuclein from the tissue sections of patients with Lewy body disease; 2) mice without EP2 were more resistant to neurotoxicity induced by MPTP, and this effect was seen in association with attenuated formation of aggregated α-synuclein in the SN and striatum; and 3) EP2^-/- ^microglia exposed to aggregated α-synuclein appeared to have less membranous translocation of p47-phox, a critical process leading to PHOX activation.

The observation that EP2^-/- ^mice had significantly increased ability in clearing aggregated α-synuclein from human tissue with Lewy body disease is identical to the observation made earlier by our group, where EP2^-/- ^microglia cleared aggregated Aβ [10] more effectively than WT microglia in human hippocampal slices. Although the precise mechanisms underlining the enhanced phagocytosis in EP2^-/- ^microglia remain to be defined, this observation is quite significant. This is because we have recently observed that aggregated α-synuclein activates microglia efficiently, leading to enhanced DAergic neurotoxicity [9]. It should also be noted that α-synuclein can be actively secreted by neurons to extracellular space where it aggregates faster [20]. Finally, our ongoing study has further illustrated that internalization of aggregated α-synuclein is not necessary for aggregated α-synuclein to activate microglia (not shown), meaning that increased phagocytosis does not necessarily translate into increased microglial activation with production of neurotoxic species [21,22]. On the other hand, activated microglia may produce neuroprotective factors [23], i.e. the final outcome of microglial activation may depend on the delicate balance between these two forces [24]. To this end, subtypes of PGE_2 _receptors may play a major role. This is because PGE_2 _can interact with four distinct receptor subtypes: EP_1_, EP2, EP_3 _and EP_4_, that are linked to functionally antagonistic second messenger systems [25]. For instance, EP_1 _increases intracellular concentration of calcium; EP2 and EP_4 _activate adenylyl cyclase via stimulatory GTP-binding proteins, while EP_3 _mainly inhibits adenylyl cyclase via inhibitory GTP-binding proteins [26]. All EP receptor subtypes are expressed on varying cells in the brain and it has been demonstrated that microglia express both EP_1 _and EP2 [27].

It is possible that ablating EP2 may have indirect effects on phagocytosis through compensatory up-regulation of other EP receptors on microglia. Ideally, a direct effect of EP2 on phagocytosis could be evaluated by restoring EP2 function in cultured EP2^-/- ^microglia. While the EP2 agonist butaprost (or CAY10399) has been a valuable tool for distinguishing EP2-specific activity [28-30], it unfortunately is of limited use in characterizing EP2^-/- ^cells, since it does not restore EP2 function in the absence of the receptor. Indeed, negative results following butaprost exposure in EP2^-/- ^mice have been used to identify EP2-specific effects, since butaprost does not bind appreciably to any of the other EP receptors [29]. For instance, Kennedy et al. demonstrated that while butaprost infusion has a significant effect on blood pressure in wt mice, no response is seen in EP2^-/- ^mice, while a prostacyclin receptor antagonist elicits a similar response in both [13].

In our MPTP model, when mice were treated with either subchronic or chronic regimen, the data clearly showed that mice without EP2 had greater striatal DA levels, the loss of which is a widely accepted marker of toxicity to DA neurons. More importantly, EP2^-/- ^mice also demonstrated less NP40-insoluble aggregated α-synuclein in both SN and striatum. As abundant evidence has suggested that aggregated α-synuclein is toxic to neurons, it is reasonable to suggest that the mechanisms by which EP2^-/- ^mice became more resistant to MPTP were at least partially attributable to their enhanced ability to either prevent the formation of or better clear aggregated α-synuclein by microglia. That being said, mechanisms other than aggregated α-synuclein may be involved in DAergic neurodegeneration, which may or may not relate to α-synuclein aggregation directly. These may include: mitochondrial inhibition [31], increased oxidative stress [32], and decreased proteasomal and lysosomal functions [33,34]. This issue is further complicated by the fact that EP2 is also expressed in cells other than microglia [35], raising the possibility that protective effects seen in EP2^-/- ^may be related to factors involving other cells. Nonetheless, the fact that microglia derived from EP2^-/- ^mice clearly showed increased capacity in the clearance of both Aβ and α-synuclein without increasing microglial paracrine neurotoxicity strongly emphasizes the role of EP2 in neurodegeneration in both AD and PD.

With respect to the mechanisms underlying microglial activation induced by α-synuclein aggregates, our *in vitro *data unequivocally showed that EP2^-/- ^microglia had less translocation of p47-phox subunit of PHOX after exposure to aggregated α-synuclein. It is known that α-synuclein is intimately associated with increased ROS production by PHOX activation [9] and that PHOX is a membrane-associated enzyme that generates O_2_^- ^by catalyzing the transfer of electrons from NADPH to molecular oxygen. The production of O_2_^- ^was also measured in microglia generated from both EP2-/- and BALB/c mice after treatment with aggregated α-synuclein (not shown). While we have measured O_2_^- ^in C57Bl/6 mice [9], in Balb/C mice the results were inconclusive as basal levels of O_2_^- ^were highly variable; a definitive result would have strengthened our interpretation of the p47 translocation data. Nonetheless, since translocation of several cytoplasmic subunits to the membranous compartment is critical to microglial activation [36,37], it is expected that ROS production in EP2^-/- ^microglia should also be attenuated as compared to WT controls after exposure to aggregated α-synuclein. Increasing evidence has indicated that p47-phox plays a central role in the assembly process of PHOX, possibly by sensing the activation signal through multiple phosphorylations and then acting as a scaffolding protein for translocation and assembly of the subunits of PHOX [38]. What remains to be studied is: why was the translocation of p47-phox, but not p67-phox, affected by ablating EP2 receptor? To this end, one of the potential fruitful areas in the further research could be cAMP-dependent signal transduction pathways. This is because many investigators have demonstrated that EP2 regulates the intracellular levels of cAMP [25,39,40], which has a significant (and controversial) effects on the translocation/activation of PHOX [41-43].

## Conclusion

In summary, we have tested our hypothesis that the EP2 receptor is critical in regulating aggregated α-synuclein levels in PD, thereby influencing neurodegeneration induced by aggregated α-synuclein via *ex-vivo*, *in vivo *and *in vitro *studies, respectively. Our results demonstrated that EP2^-/- ^microglia exhibited enhanced capacity in clearing aggregated α-synuclein in human mesocortex tissue with Lewy body disease. In addition, EP2^-/-^, while exhibiting less aggregated α-synuclein, were also more resistant to neurotoxicity induced MPTP. Finally, EP2^-/- ^microglia appeared to have less translocation of a critical cytoplasmic subunit (p-47-phox) of PHOX to the membranous compartment after exposure to aggregated α-synuclein. Further characterization of the role of EP2 receptor could lead to better understanding of the pathophysiology involved in synucleinopathy as well as the development of novel therapeutic targets that enhance microglial phagocytosis of α-synuclein aggregates while also suppressing microglia-mediated neurotoxicity.

## Competing interests

The author(s) declare that they have no competing interests.

## Authors' contributions

JJ performed experiments and drafted the manuscript. FSS, JL, YW and JD performed experiments. AMS maintained the mouse line that was used in the study. KSM assisted in data analysis and manuscript preparation. TJM and JZ conceived the study and its design and helped to draft the manuscript.
